# Chitosan-Coated Liposome Formulations for Encapsulation of Ciprofloxacin and Etoposide

**DOI:** 10.3390/pharmaceutics16081036

**Published:** 2024-08-02

**Authors:** Rubén Gil-Gonzalo, D. Alonzo Durante-Salmerón, Saeedeh Pouri, Ernesto Doncel-Pérez, Andrés R. Alcántara, Inmaculada Aranaz, Niuris Acosta

**Affiliations:** 1 Pluridisciplinar Institute, Complutense University of Madrid, Paseo Juan XXIII, 1, E-28040 Madrid, Spain; rubgil@ucm.es (R.G.-G.); saeedepo@ucm.es (S.P.); 2 Department of Chemistry in Pharmaceutical Science, Pharmacy Faculty, Complutense University of Madrid, Plaza de Ramón y Cajal s/n, E-28040 Madrid, Spain; ddurante@ucm.es (D.A.D.-S.); andalcan@ucm.es (A.R.A.); 3 Neural Regeneration Group, Hospital Nacional de Parapléjicos (SESCAM), E-45071 Toledo, Spain; ernestod@sescam.jccm.es

**Keywords:** chitosome, CS-coated liposomes, chitosan, liposome, ciprofloxacin, etoposide, thin-film hydration, glioblastoma, U373, *Escherichia coli*

## Abstract

Cancer and bacterial infections rank among the most significant global health threats. accounting for roughly 25 million fatalities each year. This statistic underscores the urgent necessity for developing novel drugs, enhancing current treatments, and implementing systems that boost their bioavailability to achieve superior therapeutic outcomes. Liposomes have been recognised as effective carriers; nonetheless, they encounter issues with long-term stability and structural integrity, which limit their pharmaceutical applicability. Chitosomes (chitosan-coated liposomes) are generally a good alternative to solve these issues. This research aims to demonstrate the effective individual encapsulation of ciprofloxacin (antibacterial, hydrophilic) and etoposide (anticancer, hydrophobic), within chitosomes to create more effective drug delivery systems (oral administration for ciprofloxacin, parenteral administration for etoposide). Thus, liposomes and chitosomes were prepared using the thin-film hydration technique and were characterised through ATR-FTIR, Dynamic Light Scattering (DLS), zeta potential, and release profiling. In both cases, the application of chitosomes enhanced long-term stability in size and surface charge. Chitosome-encapsulated ciprofloxacin formulations exhibited a slower and sustained release profile, while the combined effect of etoposide and chitosan showed heightened efficacy against the glioblastoma cell line U373. Therefore, coating liposomes with chitosan improved the encapsulation system’s properties, resulting in a promising method for drug delivery.

## 1. Introduction

The global world objective addresses a challenge in the field of “health, demographic change, and wellness”. The resistance to antibiotics is one of the greatest threats to global health, as the number of infections whose treatment becomes more difficult due to the loss of efficacy of antibiotics is exponentially increasing. This phenomenon has been recognised worldwide as the “antibiotic crisis”, and, nowadays, around 700,000 people die of resistant infections every year. Experts estimate that this will lead to a death increase of about 10 million people and, in addition, to a raise in the medical expenditure reaching 1012 million USD in the next 30 years [1]. On the other hand, cancer constitutes one of the main causes of hospital admission (approx. 3 million stays), only behind circulatory and respiratory diseases (World Health Organization statistics, 2018). Even more, cancer is the second cause of death globally, and the number of deaths caused by tumours is estimated to increase more than 50% in the year 2035. Specifically, according to a report of the Spanish Society of Oncology in 2017, the estimated new cases of cancer in Spain were 228,482, and by 2035 it is estimated to raise up to around 315,000 cases [2]. In this context, ongoing research could contribute to the knowledge of these diseases and contribute to an improvement in the quality of life of affected people.

Ciprofloxacin is a hydrophilic antibiotic belonging to the fluoroquinolone group, with a high spectrum of action, acting both on Gram-positive and Gram-negative bacteria. Ciprofloxacin is one of the most common drugs in clinical use for the treatment of several infections [3]. However, by action of lysosomes, this drug only reaches 52% bioavailability [4], so that a better protection of the drug would be necessary to improve its effectivity allowing a reduction in the drug’s dose.

Etoposide is a hydrophobic antineoplastic drug used to treat solid tumours [5,6], such as glioblastoma, a type of brain tumour that originates from glial cells, with a high proliferative potential and poor prognosis [7]. Etoposide has been used for antiglioma therapy by direct administration [8]; however, its application requires the use of different excipients to improve its solubility due to its high lipophilicity. These excipients are associated with hypotension, anaphylaxis, bronchospasm, and other adverse effects [9]. Therefore, a more effective liberation profile and a lipophilic carrier are necessary to avoid the use of excipients, which could compromise patients’ health.

Liposomes are vesicular nanoparticles generated from a lipid bilayer [10]. These particles separate an internal and an external environment by means of this membrane, thus allowing the encapsulation of both hydrophilic and hydrophobic active ingredients. This enables the protection of various molecules from chemical and biological degradation [11]. Their lipid composition and aqueous interior give liposomes an amphipathic character. In addition, their use prolongs the circulation time of pharmaceutical products showing low viability inside the human body, which may be hindered because of stability, solubility, and toxicity problems. Recent reviews have showcased the use of liposomes for antimicrobial and antitumoral applications, moving from lab-scale advancements to novel commercial formulations currently in the market [12,13].

Depending on the production process, liposomes can possess different structures, classified according to the number of layers (known as lamellae) and their distribution. When there is more than one lamella, liposomes are classified as multilamellar vesicles (MLVs) or multivesicular vesicles (MVVs), depending on whether they are concentric or non-concentric. When the liposomes consist of only one lamella, they are named unilamellar vesicles (ULVs), which are subsequently classified based on their size into: giant (GULVs) if their diameter is larger than 1000 nm, large (LUVs) if their diameter is between 100 and 1000 nm, or small (SUVs) when they have a diameter smaller than 100 nm [14].

However, liposomes aggregate and fuse over time [15]. Additionally, they can be easily degraded by changes in pH, enzymatic action, and the patient’s immune system [16]. To overcome these limitations, modification of the liposome structure by interaction with polymers has been proposed to improve the physicochemical properties of liposomes. The main advantages of polymeric NPs include high temperature and pH stability, sustained and controllable release of an encapsulated drug, ease of surface modification with ligands, aptamers or hydrophilic polymers, non-immunogenicity, reduced renal filtration rate, and ability to avoid immune system clearance [17]. Among natural polymers, chitosan has been proposed for optimisation of liposomal nanoparticles.

Chitosan (CS) is an amino-polysaccharide composed of β (1→4) linked units of *N*-acetyl-D-glucosamine (GlcNAc) and D-glucosamine (GlcN). This polysaccharide is obtained by the extensive alkaline deacetylation of chitin [18]. Depending on the natural source of chitin and the followed methodology, the obtained chitosan samples can present different degrees of acetylation (DA), homogeneous (in random) or heterogeneous (in block) acetyl distribution, and different molecular weight averages, which are physical parameters that will determine their functional properties [19]. Chitosan showcases a plethora of desirable biological properties such as antimicrobial, antioxidant, anti-inflammatory, and antitumoral activities [20]. CS is highly processable, and depending on the intended application, it can adopt different shapes such as fibres, powders, films, sponges, beads, solutions, gels, and capsules [21]. For all these reasons, and also because of other key properties such as its biodegradability, biocompatibility, mucoadhesivity, non-allergenicity and non-toxicity, chitosan is of great interest in many fields such as biomedicine, cosmetics, biotechnology, food industry, nanotechnology, etc. [22].

CS-coated liposomes, also known as chitosomes, exhibit not only a higher stability over time compared to liposomes, but also a higher bioavailability of the encapsulated compound, an increased flexibility, and a more homogeneous surface area. These qualities make CS-coated liposomes a promising encapsulation system for drugs displaying side effects and/or low bioavailability. Some authors have reported the use of chitosomes for antimicrobial and antitumoral applications [23,24,25,26,27,28,29,30].

Thus, CS-coated liposomes can be employed to increase the efficacy of antimicrobial and antitumoral agents. This has led to the possibility to further explore the use of these nanocarriers, with the objective of eventually reaching their commercialisation. For the purposes of this study, the encapsulation of a hydrophilic antimicrobial agent (Ciprofloxacin, CFX), and a hydrophobic antitumoral agent (Etoposide, Eto) was analysed.

In regard to chitosan and chitosan derivatives, how exactly these polymers exert their antimicrobial activity is still under discussion. This fact can be explained taking into account the lack of an appropriate polymer characterisation, purity issues, the use of different microorganisms, and the lack of methodological uniformity. Nonetheless, the leading hypothesis states that chitosan’s antimicrobial activity stems from the reduction in bacterial cell membrane permeability due to polymer coating on the surface of the cells, therefore blocking cell access to nutrients [31]. Coupling chitosan’s innate antimicrobial activity with ciprofloxacin could lead to a synergistic behaviour for enhancing the antibacterial efficacy, all while improving its encapsulation in liposomal nanocarriers. 

Concerning the antitumoral activity of chitosan, it should be noted that there are many reports dealing with the anticancer activity of chitosan, and this mechanism is related to both membrane-disrupting and apoptosis-inducing activities [32]. In particular, it has been shown that CS activates caspases 3 and 8, involved in the apoptotic pathway, conferring CS antitumoral activity. Coupling this innate antitumoral activity with anticancer agents can increase their efficacy, all while diminishing the potential adverse events that could be related to the use of conventional anticancer therapies. 

Taking all these factors into account, liposomes have been used to encapsulate ciprofloxacin (intended for oral administration) and etoposide (parenteral administration) to overcome any problems related to conventional dosage regimens and increase therapeutic efficacy. But, as previously mentioned, liposomes do not have a good long-term stability. Thus, an improvement in its characteristics is necessary to obtain an optimal carrier for both hydrophilic and hydrophobic drugs. In this study we used chitosan to obtain CS-coated liposomes and separately encapsulate the antimicrobial ciprofloxacin and the antitumoral etoposide, improving stability, action, and release of both drugs, for oral and parenteral delivery, respectively.

## 2. Materials and Methods

### 2.1. Materials

Chitosan (CS) Chitopharm™ S was purchased from Chitinor AS (Tromsø, Norway) (77% deacetylated; Mw 91.5 kDa). *L*-α-Dipalmitoyl phosphatidylcholine (DPPC) was purchased from Sygena. Ciprofloxacin (CFX), etoposide (Eto), lanolin cholesterol (Chol) (purity ≥ 99.0%), phosphate buffer saline (PBS) tablets, dialysis bags (Spectra/Por membrane, Mw cutoff = 14 kDa), and dichloromethane (DCM) were acquired from Sigma-Aldrich (Sigma-Aldrich, Saint Louis, MO, USA). Chloroform and methanol (MeOH) were acquired from PanReac (PanReac AppliChem GmbH, Darmstadt, Germany). All other chemicals were used without further purification.

### 2.2. Methods

#### 2.2.1. Liposome Preparation for Hydrophilic Drug Encapsulation

Liposomes were synthesised following a modification of the thin-lipid film hydration method described by Rosales-Mendoza and González-Ortega [33]. Briefly, 29.36 mg of DPPC was dissolved in 4 mL of DCM. Then, 2 g of glass pearls were added, and the organic solvent was evaporated in a rotary evaporator at 41 °C, 800 mbar (relative pressure), and 35 rpm (R-210, Büchi Labortechnik, Flawil, Switzerland). Once the film was formed, the pressure was reduced to 100 mbar for 2.5 h. To rehydrate the film, 4 mL of 0.3 mg/mL CFX water solution was added to the evaporation flask and placed again in the rotary evaporator at 51 °C, 55 rpm, and atmospheric pressure for 1 h [34]. In this experiment, the flask was transferred to an ultrasonic bath (JP Selecta, Barcelona, Spain) every 10 min. It was then exposed to 30 s of ultrasound at 40 kHz and 120 W to prevent particle aggregation. After 30 min and 1 h, the flask underwent a 3-min ultrasound treatment to further homogenise the particles. The resulting liposomes (CFX-SUVs) were stored at 4 °C until use. 

#### 2.2.2. Liposome Preparation for Hydrophobic Drug Encapsulation

Liposomes were synthesised following a variant of the previously described method for obtention of SUVs. For this purpose, 14.81 mg of the DPPC and 0.84 mg of cholesterol were dissolved in CHCl_3_/MeOH (7:3, 4 mL) containing 0.2 mg/mL of Eto. Then, 2 g of glass pearls were added, and the organic solvent was evaporated in a rotary evaporator at 41 °C, 200 mbar (relative pressure), and 35 rpm. Once the film was formed, the pressure was reduced to 10 mbar for 4 h. To rehydrate the film, 2.5 mL of distilled water was added to the flask and placed again in the rotary evaporator at 51 °C, 55 rpm, and atmospheric pressure for 1 h. The homogenisation procedure was conducted using the same established method for encapsulating hydrophilic drugs. The resulting liposomes (Eto-CholSUVs) were stored at 4 °C until use.

#### 2.2.3. CS-Coated Liposomes Preparation

CS-coated liposomes were prepared from liposomes according to Haeri et al. [35]. To 1 mL of liposome solution, 1 mL of CS solution (0.6%, *w*/*v* in 0.1 M acetic acid) was added dropwise and the mixture was left under magnetic stirring for 1 h. Finally, the mixture was kept at 4 °C for 16 h to complete the CS-coated liposomes formation. The different sample composition is summarised on Table 1.

#### 2.2.4. Characterisation of Synthesised Particles

##### ATR-FTIR Analysis

CFX, Eto, DPPC, empty liposomes, empty CS-coated liposomes, CFX-SUVs, CFX-SUVs-CS, Eto-CholSUVs, and Eto-CholSUVs-CS samples were lyophilised (LyoQuest, Azbil Telstar, Tokyo, Japan) and analysed in an Agilent Technologies Cary 630 FTIR (Agilent, Santa Clara, CA, USA). The spectral resolution was 4 cm^−1^ with 64 scans with a range of 600 to 4000 cm^−1^.

##### Particle Size and Zeta Potential Measurements

Particle size and particle size distribution of liposomes and CS-coated liposomes were determined via Dynamic Light Scattering (DLS) technique using Malvern Zetasizer 3000 HS (Malvern Instruments, Malvern, UK) at a 900-scattering angle. An aliquot of each sample was diluted 8-fold with NaCl 1 mM and the size was determined after thorough mixing of samples. 

Zeta potential of the undiluted liposomes and CS-coated liposomes was determined using Malvern Zetasizer 3000 HS (Malvern Instruments). Zeta potential was calculated by Smoluchowski’s equation from the electrophoretic mobility [36] of liposomes at 25 °C. 

The average particle size and zeta potential were measured in triplicate and average values were calculated.

#### 2.2.5. Encapsulation Efficiency and Drug Loading

Entrapment efficiency (%) was determined using the centrifugation method. The sample were centrifuged at 9500 RCF for 10 min (Heraeus Pico 17, ThermoScientific, Waltham, MA, USA). The separated supernatant was collected in separate vacutainers and stored at 4 °C for further analysis. 

High-performance liquid chromatography (HPLC) Agilent 1260 Infinity II with a C18 column Agilent InfinityLab Poroshell 120 (Agilent, Santa Clara, CA, USA) was used for the determination and quantification of both drugs (CFX and Eto). The mobile phase combined two eluents (A:B) in a 75/25 ratio at a flow rate of 1 mL·min^−1^, with detection at 210 nm. Eluent A: acetonitrile:water:Formic Acid (95:4.5:0.5 *v*/*v*/*v*); eluent B: acetonitrile:water:Formic Acid (4.5:95:0.5 *v*/*v*/*v*). 

A calibration curve was accordingly prepared for both compounds with elution times of 12.4 mts (CFX) and 45.5 mts (Eto), respectively. Chromatograms were analysed at 210 nm, and drug concentrations were calculated using previously developed standard calibration curves. Thus, the amount of drug entrapped (entrapment efficiency) inside the liposomes and CS-coated liposomes were calculated by subtracting the values in the supernatant from the initial concentrations. 

#### 2.2.6. In Vitro Drug Release

Two different methods were used to study the in vitro release, depending on the studied drug. Release studies were conducted in triplicate using the dialysis method; 1 mL of the formulations was added to dialysis bags and placed in a determined volume of the release medium at 37 °C and 100 rpm. At predetermined time points, a specified sampling volume of the release medium was withdrawn from each dissolution flask and replaced by fresh medium.

The release behaviour of CFX was evaluated in 25 mL of Simulated Gastric Fluid (SGF), which consisted of 0.1 M HCl and 2.9 g/L NaCl, with a pH of 2. This was conducted for a duration of 2 h. During sampling, 1 mL of the release medium was taken in order to measure CFX concentration at 210 nm using a spectrophotometer (NanoDropOneC, ThermoScientific). After each sampling, 1 mL of fresh SGF was added to replace the volume that was retrieved. The release profile of Eto was examined in 25 mL of 0.1 M PBS at a pH of 7.4 for a period of 24 h. The measurement was carried out using HPLC based on the previously outlined method. As a complementary method, etoposide release studies were determined at 285 nm by UV-vis spectrophotometry (NanoDropOneC, ThermoScientific). 

The in vitro release profiles of selected drug-loaded liposomes and CS-coated liposomes dispersions were plotted to estimate the release kinetics of the developed formulations. Results were then fitted to the first order, Higuchi, Baker–Londsdale, and Weibull kinetic models; then, the Korsmeyer–Peppas mechanistic model was used to determine the release mechanism. As the obtained constructions are spherical, the diffusion exponent was set for spherical shape [37]. The relation between n (diffusional exponent) value and the release mechanism is summarised in Table 2.

#### 2.2.7. Antimicrobial Activity of CFX-SUVs and CFX-SUVs-CS

The bacterial inoculums were prepared following the guidelines of the Clinical and Laboratory Standards Institute (CLSI) [38]. The bacterial cultures were mixed with Luria Bertani (LB) and their turbidity was adjusted to match the 0.5 McFarland turbidity standards. The agar plate method was used to check the antimicrobial activity of the chitosan. A LB (0.1 mL) broth culture of the *E. coli* ATCC 25922 was spread over Mueller–Hinton Agar (MHA) plates. Afterward, solutions containing CFX-SUVs, CFX-SUVs-CS, and CS were cultured on the plate and using CFX as a positive control with a final concentration of 0.15 µg/mL. The plates incubated at 37 °C for 24 h.

#### 2.2.8. In Vitro Cytotoxicity Studies of Eto-CholSUVs and Eto-CholSUVs-CS

The cytotoxicity of etoposide-containing liposomes and CS-coated liposomes was evaluated in C166-GFP mouse endothelial cell line (ATCC CRL2583™, Manassas, VA USA) through an analysis of their metabolic activity. The cells were cultured in a 24-well plate, with a cell density of 30,000 cells per well in Dulbecco’s Modified Eagle Medium (DMEM) supplemented with 10% Foetal Bovine Serum (FBS) and streptavidin/biotin as antibiotic. The plate was incubated at 37 °C and 5% CO_2_ and after 48 h, different concentrations of free etoposide and etoposide-containing liposome/CS-coated liposome solution were added. 

After 24 h, the cell’s metabolic activity was evaluated using the Alamar Blue assay, following the instructions of the manufacturer (Biosource, Camarillo, CA, USA). For this, 100 µL of the Alamar Blue dye were added to each well containing living cells and incubated for 90 min at 37 °C with 5% CO_2_. Then, the fluorescence of each well was measured using a Synergy HT plate reader (BioTek, Winooski, VT, USA) at 535/590 nm.

#### 2.2.9. Cell Growth Inhibition

The human glioblastoma cell line U373-MG was used for cell growth inhibition assays. Cells were seeded in DMEM plus 10% FCS at 1 × 10^4^ cells/well and allowed to attach for 6 h. The medium was changed to serum-free DMEM, and the cells were incubated for 36 h, then the medium was replaced with DMEM plus 2% FCS, containing the encapsulated etoposide inhibitors or controls, and incubated for 72 h. The medium was carefully aspirated and MTT reagent (Sigma) was added to the cell culture and incubated for 4 h. Cells were lysed overnight with lysis solution (10% SDS, 0.01 M HCl). Absorbance was measured in a microplate reader at 570 nm. The cell growth inhibition of U373-MG cells was calculated as follows:

% inhibition = 100 − 100 [(X − B)/(A − B)](1)

where A is the mean experimental absorbance (Abs) corresponding to cells maintained in DMEM plus serum, B is the mean experimental Abs of cell-free medium, and X corresponds to experimental Abs from cells treated with etoposide inhibitors or control variants. Each treatment was assessed in triplicate. GraphPad Prism version 8 software was used for graphs and statistical analysis.

#### 2.2.10. Statistical Analysis

Results for tissue distribution studies were expressed as mean S.D. of three experiments. Statistical comparison of data was performed via ANOVA at a significance level of *p* < 0.01 and Student’s *t* test at *p* < 0.001. 

## 3. Results and Discussion

In this paper, a hydrophobic drug (etoposide, Eto) and a hydrophilic drug (ciprofloxacin, CFX) were encapsulated in liposomes and CS-coated liposomes to study their viability as drug carriers.

The encapsulation of CFX was intended for oral delivery, and chitosan-coated liposomes are expected to improve the delivery in this route due to a stabilisation of the liposomes [39]. On the other hand, Eto encapsulation was designed for a parenteral route, aiming to increase the antitumoral effect by enhancing the drug intake by tumoral cells [40].

### 3.1. ATR-FTIR Analysis

**Figure 1:**
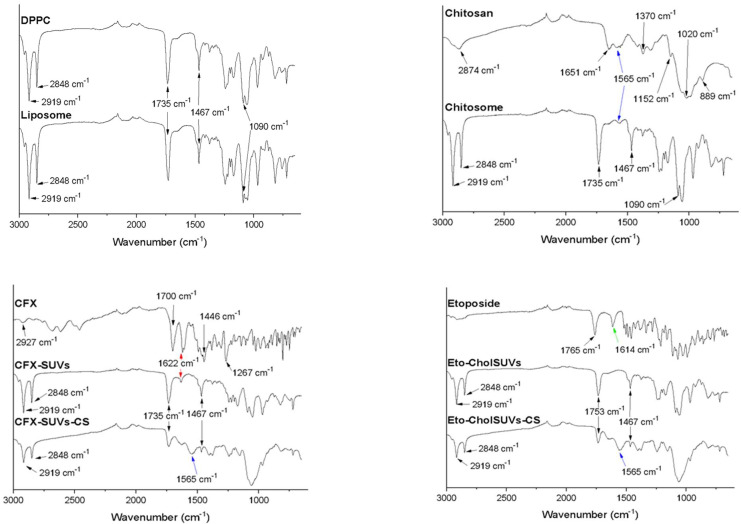
Compared ATR spectra. Red arrow: 1632 cm^−1^ band of CFX. Green arrow: 1614 cm^−1^ of Eto. Blue arrow: 1565 cm^−1^ band of CS.

The ATR-FTIR spectra of DPPC, CFX, Eto, empty liposomes, empty CS-coated liposomes, CFX-SUVs, CFX-SUVs-CS, Eto-CholSUVs, and Eto-CholSUVs-CS are shown in Figure 1.

The DPPC spectrum shows CH_2_ stretching vibration peaks at 2919 and 2848 cm^−1^, and a CH_2_ scissoring vibration peak at 1467 cm^−1^. Peaks for C=O and PO_2_^−^ vibrations are at 1735 and 1090 cm^−1^, respectively [41]. As the liposome’s spectrum is the same as the DPPC’s spectrum during the synthesis method, no modification was made to the chemical structure of DPPC.

When adding the CS coating to liposomes, small changes can be seen in the spectrum. The amount of CS used (0.3%) for coating is low and the main difference between liposomes as CS-coated liposomes is the presence of a small band observed at 1565 cm^−1^, attributed to N-H vibrations of the deacetylated groups of CS [42]. This band is also visible in CFX-SUVs-CS and Eto-CholSUVs-CS. Typical CH vibrations of CS appear at 1370 and 2874 cm^−1^. The saccharide structure of CS produces peaks at 889 and 1152 cm^−1^; the C=O stretching vibrations produces a broad peak at 1020 cm^−1^ [43].

The CFX spectrum illustrated in Figure 1 shows characteristic absorption bands at 1267 and 1622 cm^−1^, thus indicating the stretching vibration of the C-F bond and the vibration of the phenyl framework conjugated to –COOH, respectively [44]. The stretching vibrations in 1700 and 1446 cm^−1^ are attributed to –COOH and C-H bending, respectively [45,46]. The stretching vibration of C-H from the phenyl framework of CFX is visible at 2927 cm^−1^ [47,48]. 

The employed CFX shows a typical C=O vibration band at 1622 cm^−1^ [46]. This band is also visible in CFX-SUVs but not in CFX-SUVs-CS. As ATR-FTIR measures the active groups on the surface of the samples [49] and CFX is a hydrophilic drug which is entrapped in the aqueous core of the liposome, the presence of this band represents the non-encapsulated drug in CFX-CS. Meanwhile, in CFX-SUVs-CS, the N-H vibration of the deacetylated groups of CS overshadows the C=O vibration of non-encapsulated CFX.

Etoposide has two easily distinguishable bands, one produced at 1765 cm^−1^ by the lactone group and the other at 1614 cm^−1^ by the aromatic groups [50]. None of these two bands are present in Eto-CholSUVs, indicating that Eto is being encapsulated inside the lipid bilayer and not on its surface [51]. These results are in line with the fact that Eto is not detected in Eto-CholSUVs-CS either. It is remarkable that CFX-SUVs-CS and Eto-CholSUVs-CS show similar spectra, but this was expected, as both are mainly constituted of the two same compounds (DPPC and CS).

### 3.2. Particle Size and Zeta Potential

SUVs and CholSUVs are known for their versatility as carriers for different routes including the oral one [39], being the surface of spherical vesicles (which is directly related to their diameter), the main factor for determining the availability and absorption in this route. Another essential point is the surface charge that defines the stability, circulation time, interaction with proteins, permeability through cellular barriers, and biocompatibility of the particles [52]. Surface charge was measured through zeta potential (ζ). As stablished, for values of ζ < ±5 mV, aggregation occurs, and brief time stability is achieved when ζ ≈ ±20 mV. Good long-term stability is reported for values in the range of ±30 ≤ ζ ≤ ±60 mV [53].

Therefore, it is necessary to determine the particles’ size and charge to properly understand the characteristics of the formulations. In this study, hydrodynamic size and surface charge of the particles were determined through Dynamic Light Scattering (DLS) and zeta potential techniques, respectively.

Results for samples obtained through the hydrophilic drug encapsulation for oral drug delivery are shown in Table 3. Samples with no homogenisation process were also analysed, and the particles obtained had diameters ranging from 1000 to 4000 nm. The ultrasonication of the samples reduced the size of liposomes and chitosan-coated liposomes to 90–200 nm. The presence of CS-coating increased the diameter, as reported [54]. This effect was still visible after a week, indicating that the coating is stable over time and does not separate from the lipid bilayer. Additionally, the samples showed an increase from +25 mV to +45 mV in zeta potential when the CS-coating was present. This fact is caused by CS bonding to DPPC, therefore changing the surface charge of the vesicles [55]. Therefore, the coating with chitosan in the hydrophilic drug encapsulation method improved the long-term stability of liposomes and their colloidal stability [56]. 

In the results for the hydrophobic drug encapsulation method for parenteral drug delivery (shown in Table 4) the CholSUVs showed a diameter of 93 nm, which doubled when coating with CS (203 nm). The presence of encapsulated Eto resulted in an increase in diameter to 323 and 689 nm in Eto-CholSUVs and Eto-CholSUVs-CS, respectively. These results, when compared to SUVs and SUVs-CS, show a decrease in particle diameter from 2919 nm in SUVs-CS to 203 nm in CholSUVs-CS, due to the addition of the ultrasound treatment to the synthesis method. Also, the ultrasonication produced a decrease in PDI, demonstrating the importance of ultrasonication as a homogenising element. Nevertheless, even if the addition of the ultrasonication process decreased the PDI from 0.607 in SUVs to 0.209 in CholSUVs, the presence of a CS coating produces an increase in PDI that cannot be overcome by the single effect of ultrasonication, making necessary the use of a second homogenisation process to obtain a monodisperse distribution.

Eto-CholSUVs-CS show a bigger initial diameter than Eto-CholSUVs due to the addition of CS, but the presence of the polymer stabilises the formulations and Eto-CholSUVs-CS have little alteration in size after seven days. Even if Eto-CholSUVs-CS have a diameter of 688.5 nm, the parenteral delivery route is still available because, as their diameter is below 5 µm, they will not produce capillary blockage [57].

Contrary to what was seen for SUVs, zeta potential values for CholSUVs decreased to values lower than +30 mV with time, so that their stability was gradually declining. Nevertheless, when coating with CS, this effect was not observed, which proves their higher time-stability compared to those without CS.

Eto-CholSUVs have a bigger diameter than CholSUVs, and their size is not stable over time as deduced by a decrease in zeta potential from +26 mV to +10 mV, therefore leading to aggregation. The stabilising effect of chitosan prevents Eto-CholSUVs-CS from decreasing their surface charges, so that they do not aggregate; additionally, their size did not change after seven days, thus proving that an increased long-term stability was achieved. 

### 3.3. In Vitro CFX Release

The encapsulation efficiency of CFX-SUVs and CFX-SUVs-CS was 98 and 99%, respectively, so the coating with CS did not affect the CFX encapsulation. 

**Figure 2:**
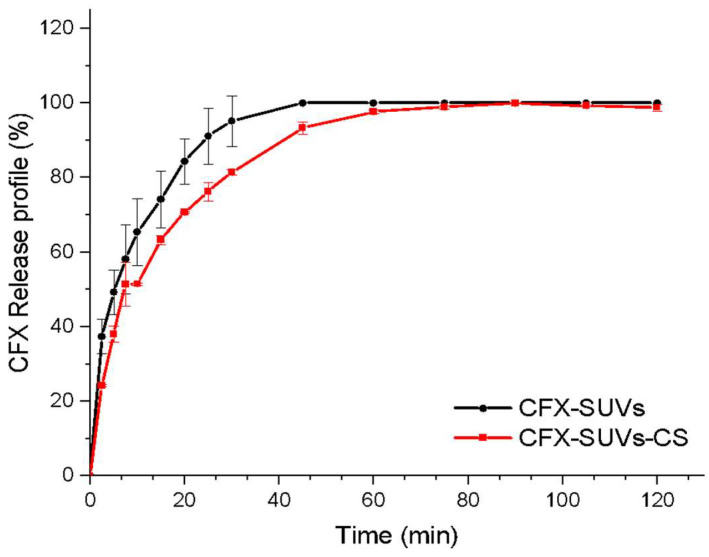
CFX release profile from CFX-SUVs and CFX-SUVs-CS in SGF.

The release study was conducted on SGF at pH 2 for 2 h (Figure 2) which simulate the stomachal environment. The CFX concentration was measured by UV-vis spectrophotometry at 278 nm (NanoDropOneC, ThermoScientific).

CFX-SUVs showed a 50% release after 5 min in the study conditions and reached 100% after 45 min (Figure 2). This is in contrast with the release profile of free CFX, which displayed a burst effect. On the other hand, the release from CS-coated liposomes was sustained in time and no burst effect was seen [29,58]. Half the encapsulated drug was released after 7.2 min, and the total of the encapsulated drug was released after 75 min instead of 45 min.

As a zwitterionic molecule, ciprofloxacin is highly soluble in pH < 5 [59], which produces a fast dissolution in gastric media. The reduction observed in the release of CFX from CFX-SUVs-CS proved to be more optimal than that obtained from CFX-SUVs. CS-coated liposomes encapsulating CFX have proven to be also more efficient than liposomes in the ocular [60] and the inhalation routes [61].

Korsmeyer–Peppas, First Order, Higuchi, Baker–Lonsdale, and Weibull models were analysed (Table 5). The Korsmeyer–Peppas mechanistic model indicates an n value of 0.4 for CFX-SUVs and 0.53 for CFX-SUVs-CS, corresponding to a hindered Fickian diffusion and an anomalous transport, respectively (see Table 2). This anomalous transport is characterised by the presence of another diffusion mechanism in addition to Fickian diffusion [62]. At low pH values, such as that one used in this study, CS swelling is high [63]. This effect produced by the CS coating, in addition to the liposome’s inherent hindered Fickian diffusion, results in the anomalous transport detected by Korsmeyer–Peppas model. Interestingly, the same cause had been previously reported to explain the anomalous transport in liposomes conjugated to other polymers [64]. 

Among the four kinetic models used, Higuchi and Baker–Londsdale showed a similar R^2^. To determine which one is the most accurate, we must consider that the Baker–Londsdale model implies a Fickian diffusion [65] but, as commented before, this is not the case; thus, the Baker–Londsdale model can be discarded, so that the release data must follow Higuchi’s model, which, on the other hand, is one of the most common models when referring to liposomes’ kinetics [66,67].

### 3.4. Antimicrobial Activity of CFX-SUVs and CFX-SUVs-CS

**Figure 3:**
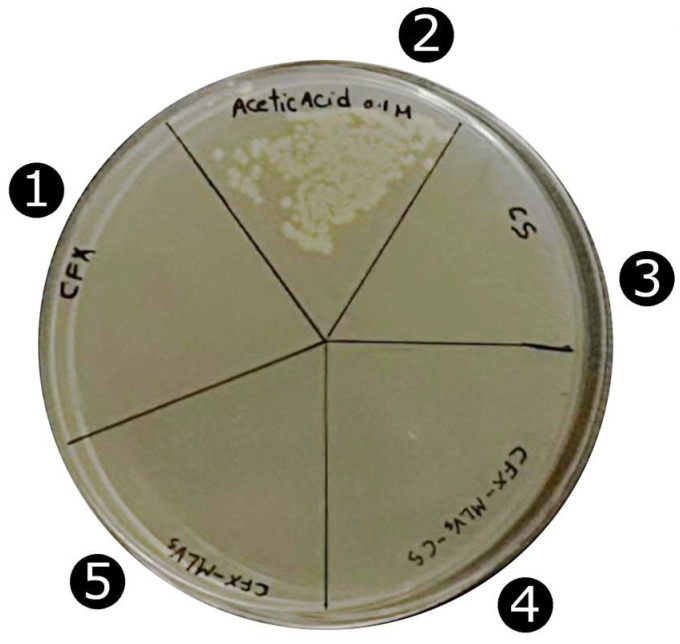
Antibacterial activity of CFX and its formulations. Ciprofloxacin (**1**), acetic acid 0.1 M (**2**), chitosan solution 0.3% (**3**), CFX-SUVs-CS (**4**), and CFX-SUVs (**5**).

Liposomes and CS-coated liposomes in solution have turbidity hindering the qualitative assessment of antimicrobial activity. Therefore, the agar-base method was used to determine the antimicrobial activity of liposomes and CS-coated liposomes loaded with CFX (Figure 3).

The test showed that there was no bacterial growth detected on CFX, CFX-SUVs, CFX-SUVs-CS, or CS. Therefore, CFX did not lose its antimicrobial effect against *E. coli* either during particle synthesis or during chitosan coating, as has been reported for *Pseudomonas aeruginosa* [68].

Chitosan and some chitosan derivatives exhibit antimicrobial activity against many microorganisms, this activity depending on the polymer’s physicochemical properties and the derivatives’ chemical nature [69]. The antimicrobial effect of CS is well-known [70,71]. In this study it has been proved that, at a very low concentration (0.3%), the effect is still present, which is useful to enhance the antimicrobial activity of CFX when encapsulated in CFX-SUVs-CS.

### 3.5. In Vitro Eto Release 

The entrapment efficiency for Eto-CholSUVs and Eto-CholSUVs-CS was found to be 94% and 96%, respectively. Thus, the CS-coating did not have a negative effect on Eto encapsulation. It has been previously described an increase in encapsulation efficiency after CS-coating liposomes containing food bioactives [72], in particular for those containing -OH groups, which showed a tendency to be more efficiently encapsulated by the effect of CS-coating due to an interaction between the -OH groups and -NH_2_ chitosan groups. A similar effect may be occurring via the etoposide’s two hydroxyl groups.

**Figure 4:**
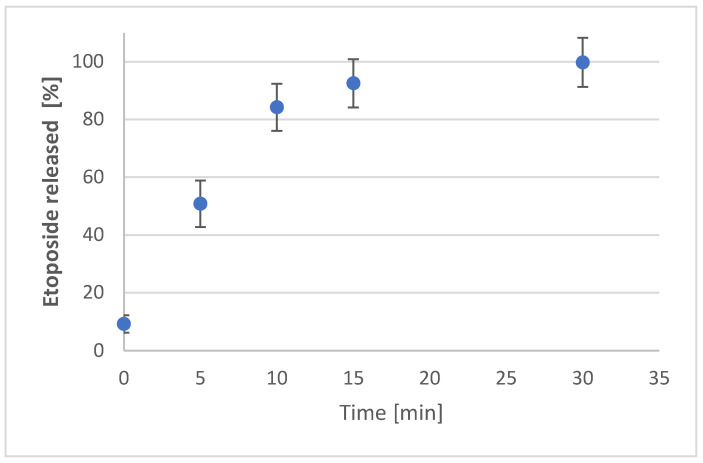
Etoposide release in 0.1 M PBS (pH 7.4).

The release study for free etoposide was conducted using a dialysis bag containing 1 mL of a 1 mg/mL etoposide containing PBS:acetonitrile solution. Initially, etoposide was dissolved in a PBS:acetonitrile (750 µL PBS:250 µL acetonitrile) mixture. The dialysis bag with the etoposide sample was placed in 10 mL of 0.1 M PBS (pH 7.4) as the release medium for 2 h. During sampling, 20 µL of the release medium was collected for spectrophotometric analysis of etoposide concentration at 285 nm. The results indicated that all the free etoposide was released within approximately 30 min, as depicted in Figure 4.

**Figure 5:**
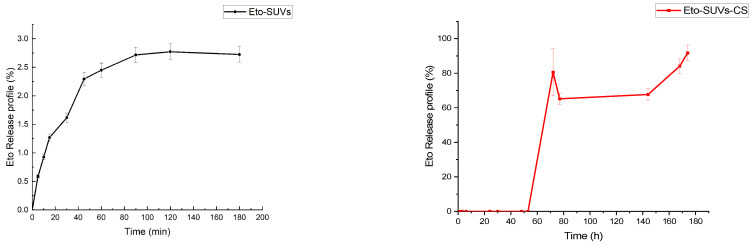
Etoposide release in 0.01 M PBS (pH 7.4) from Eto-SUVs and Eto-SUVs-CS.

The release of etoposide from the synthetised particles was performed using a dialysis bag containing 1 mL of Eto-CholSUVs or Eto-CholSUVs-CS. The dialysis bag was immersed in 25 mL of 0.01 M PBS (pH 7.4) as a release media. Release data were initially analysed at 285 nm using UV-vis spectrophotometry (Figure 5).

After 120 min in the release media, less than the 3% of the encapsulated Eto was released from the Eto-SUVs. In the case of Eto-SUVs-CS, no release was detected before 72 h of experiment. For a better understanding of the Eto release, HPLC, a more sensible technique, was used. Eto concentration was measured via HPLC at 210 nm but it was not possible to detect the drug during the 24-h study period. The limit of Eto detection was set to 0.015 μg/mL by Algan et al. [73]. This value represented the 0.2% of the encapsulated Eto in this work. Therefore, none (or less than the 0.2%) of the Eto had been released by the end of the experiment.

### 3.6. In Vitro Cytotoxicity Studies of Eto-CholSUVs and Eto-CholSUVs-CS

**Figure 6:**
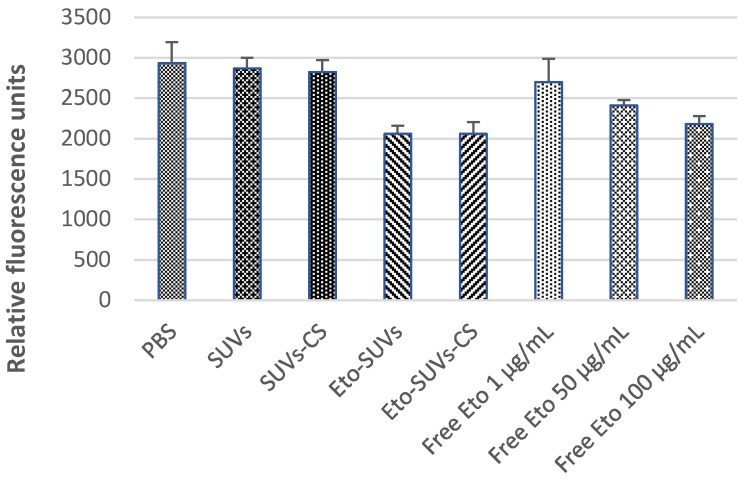
Quantification of cellular metabolic activity via the AlamarBlue technique in cells of the C166-GFP line.

The impact of Eto-CholSUVs and Eto-CholSUVs-CS on cellular metabolism was evaluated through the AlamarBlue technique. The results showed that both the free drug and the encapsulated formulations reduced the metabolic activity in mouse yolk sac endothelial cell line C166-GFP, commonly used to study cytotoxicity against the vascular epithelium. On the other hand, the empty particles did not affect the cells, showing similar measurements to cells that were not exposed to etoposide (Figure 6).

These results are in accordance with what was observed by Zare Kazemabadi and collaborators, who demonstrated that both free etoposide and etoposide-containing liposomes had a negative impact on cell viability in human lung cancer cell lines A-549 and Calu6 [74]. These preliminary results show a potential antitumoral activity from Eto-CholSUVs and Eto-CholSUVs-CS.

### 3.7. In Vitro Cell Growth Inhibition in Eto-CholSUVs and Eto-CholSUVs-CS

**Figure 7:**
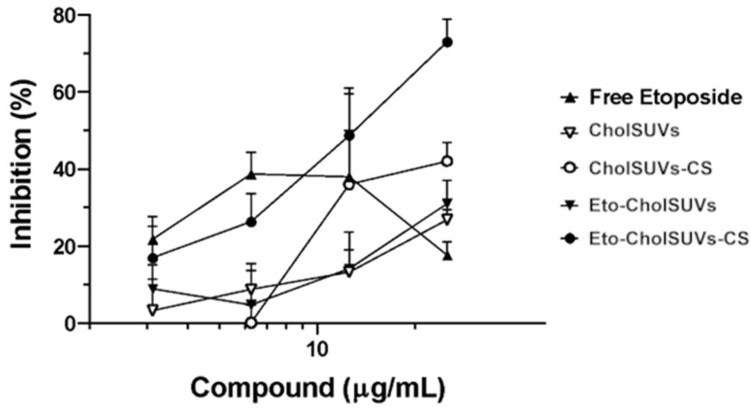
Inhibitory effect of etoposide formulations against U373 cell line, showing free etoposide in PBS, CholSUVs, CholSUVs-CS, Eto-CholSUVs, and Eto-CholSUVs-CS.

The cytotoxicity effect of etoposide on glioblastoma cell line U373 showed a saturation-like profile (Figure 7), having a maximum inhibitory activity between 6 and 13 µg/mL. From this point onwards, as the Eto concentration increases, the inhibitory activity is strongly reduced. The reduction in drug effect at high concentrations is usually caused by the action of a drug efflux pump such as the MRP1 protein, which works as a pump capable of expelling a large number of drugs from the cell, including etoposide [75]. This mechanism involved in drug resistance has been described in U373 cell line [76,77] so the reduction in growth inhibition can be attributed to this mechanism. 

The inhibitory effect of Eto-CholSUVs was expected to be higher than CholSUVs because extensive studies have reported that liposomes increase the efficacy and reduce the toxicity of antineoplastic agents [78]. Previous studies have also demonstrated that encapsulation in cationic liposomes enhanced the efficacy and reduced the toxicity of etoposide [9]. It was suggested that these effects could be explained attending to the size of the liposomes, leading to lesser opsonisation by the reticulo-endothelial cells, or because of the enhanced permeability and retention effect (EPR phenomenon) as described by Matsumura and Maeda [79]. Yet, no differences were observed between CholSUVs and Eto-CholSUVs, and therefore it can be concluded that the encapsulated Eto was not being released or taken up by the cells. This result is in line with the data obtained for the in vitro Eto release where low concentrations of Eto were released. The lack of uptake could be explained due to the brief time stability detected by zeta potential on Eto-CholSUVs (+26 mV). This could lead to aggregation and precipitation events that would hinder drug uptake by cells. For a greater inhibitory effect of Eto-CholSUVs, it would be necessary to use a higher concentration or use a drug mix like etoposide and docetaxel [80] for a combined effect or increase the surface charge by coating the liposomes with a charged polymer as the one used in this study.

Considering the previous results, liposomes were coated with a positive-charged polymer (Eto-CholSUVs-CS). This new formulation showed higher values in zeta potential (+37 mV), therefore avoiding aggregation and precipitation problems. We observed a cell growth inhibition close to 80%, and no effect from the drug efflux pump was seen since no decrease in inhibition was observed as the drug concentration increased. Other authors have also observed that an antiglioma agent encapsulated in delivery systems coated with chitosan had a more effective antitumoral capacity than the free antitumoral agent [81]. Several facts need to be considered to explain the improvement in the inhibitory effect in U373 of Eto-CholSUVs-CS formulation compared to free etoposide.

Firstly, it is known that CS activates caspases 3 and 8, involved in the apoptotic pathway, conferring CS antitumoral activity [82]. Secondly, Eto effect is hindered by its lack of solubility and stability in solution [83]. The high adhesiveness property of chitosan to cell surfaces could favour a more efficient local delivery of etoposide [84]. This is possible because chitosan coating increases the surface charge of the particles to positive values, favouring adhesion and particle internalisation [85]. Finally, etoposide’s mechanism of action is endocytosis-dependent [86]. This cellular process is related to the cell’s surface proteins, and CS has been found to have a greater interaction with those proteins due to its amine and hydroxyl groups; hence, CS-coated liposomes have a greater cellular uptake via endocytosis than liposomes [40]. 

All of the above-mentioned anticancer properties of chitosan [27,87] generate an additive effect to the antitumor capacity of etoposide against the human glioma U373 cell line.

Finally, another important factor that enhances the promising aspect of CS-coated liposomes is the reported fact showing how the penetration ability of antitumor chitosan (CS) particles into the blood-brain barrier (BBB) mainly depended on the CS component [88]. CS, as a cationic polysaccharide, can bind firmly to the cell surface and mucosa, facilitating the opening of tight junctions, thus increasing BBB penetration [89]. A better BBB penetration in addition to a higher antitumoral effect make CS-coated liposomes encapsulating Eto a promising vehicle for the treatment of glioma.

## 4. Conclusions

This study has demonstrated the ability and relevance of chitosan as a coating material for liposomes to encapsulate both hydrophilic and hydrophobic drugs, improving their availability, stabilising the drug release and showing the versatility of this system. Moreover, an encapsulation efficiency greater than 90% for both drugs and a good long-term stability were observed. Good antimicrobial activity was detected in encapsulated ciprofloxacin and an increased antitumoral effect against glioblastoma cells resulted from encapsulating etoposide in CS-coated liposomes.

## Figures and Tables

**Table 1:** Samples composition summary.

Sample	CS (%)	Cholesterol (%)	CFX (mg/mL)	Eto (mg/mL)
SUVs	-	-	-	-
SUVs-CS	0.3	-	-	-
CholSUVs	-	5.6	-	-
CholSUVs-CS	0.3	5.6	-	-
CFX-SUVs	-	-	0.3	-
CFX-SUVs-CS	0.3	-	0.3	-
Eto-CholSUVs	-	5.6	-	0.2
Eto-CholSUVs-CS	0.3	5.6	-	0.2

**Table 2:** Korsmeyer–Peppas’ diffusional exponent interpretation for spherical samples.

Release Mechanism	Hindered Fickian Diffusion	Fickian Diffusion	Anomalous Transport	Non-Fickian Transport	Super Case II
Diffusional exponent, n	n < 0.43	n = 0.43	0.43 < n < 0.85	n = 0.85	n > 0.85

**Table 3:** Size and zeta potential of SUVs, SUVs-CS, CFX-SUVs, and CFX-SUVs-CS.

Sample	Day	Diameter (nm)	PDI	Zeta Potential (mV)
SUVs	1	88.58 ± 0.51	0.238	+27.7 ± 0.557
	7	89.32 ± 1.12	0.236	+26.3 ± 1.51
SUVs-CS	1	194 ± 1.33	0.454	+44 ± 1.86
	7	195.8 ± 1.41	0.439	+48.9 ± 0.81
CFX-SUVs	1	87.53 ± 0.13	0.244	+24.2 ± 1.16
	7	86.03 ± 0.15	0.230	+28.6 ± 1.21
CFX-SUVs-CS	1	197.3 ± 1.26	0.477	+44.8 ± 2.5
	7	194.1 ± 1.15	0.445	+40.2 ± 1.44

**Table 4:** Size and zeta potential of CholSUVs, CholSUVs-CS, Eto-CholSUVs, Eto-CholSUVs-CS.

Sample	Day	Diameter (nm)	PDI	Zeta Potential (mV)
CholSUVs	1	93.18 ± 0.26	0.209	+30.3 ± 1.31
	7	93.58 ± 0.627	0.207	+27.6 ± 0.379
CholSUVs-CS	1	203.2 ± 2.76	0.462	+48.6 ± 0.81
	7	182.2 ± 1.973	0.410	+49.8 ± 2.12
Eto-CholSUVs	1	323.1 ± 17.73	0.781	+26 ± 0.25
	7	1309 ± 88.88	0.717	+10.7 ± 0.25
Eto-CholSUVs-CS	1	689 ± 16.72	0.638	+37.6 ± 1.27
	7	688.5 ± 26.08	0.599	+39 ± 0.7

**Table 5:** Analysed models for CFX-SUVs and CFX-SUVs-CS release profile.

Model	Equation	CFX-SUVs		CFX-SUVs-CS	
		Equation	R^2^	Equation	R^2^
Korsmeyer–Peppas	C_t_/C_T_ = k · t^n^	y = 0.404x + 3.248	1	y = 0.534x + 2.750	0.963
First Order	dC/dt = k · (C_s_ – C_t_)	y = 0.030x + 3.753	0.877	y = 0.026x + 3.571	0.744
Higuchi	C_t_/C_T_ = k · t^1/2^	y = 18.954x	0.994	y = 15.257x	0.994
Baker–Lonsdale	C_t_/C_T_ − 1,5 · [1 − (1 − C_t_/C_T_)^2/3^] = k · t	y = −0.0118x	0.997	y = −0.0068x	0.997
Weibull	Ln[ln(1/(1 − C_t_/C_T_)] = βln(t) + ln(α)	y = 0.748x − 1.577	0.970	y = 0.738x − 1.949	0.985

## Data Availability

The original contributions presented in the study are included in the article; further inquiries can be directed to the corresponding author/s.
